# Left atrial myxoma: an unusual cause of pre-syncope and symptomatic bradycardia

**DOI:** 10.1186/s12872-022-03018-5

**Published:** 2022-12-30

**Authors:** Kenny Vongbunyong, Steven Sinfield, Ned Premyodhin, Kevin Chen, Emin Zargarian, Angie Ng, Morton Kern

**Affiliations:** 1 Department of Medicine, University of California, Irvine, 333 The City Blvd. West, Suite 400, Orange, CA 92868 USA; 2 Department of Medicine, Division of Cardiology, University of California, Irvine, USA; 3 Department of Medicine, Division of Cardiology, Veterans Affairs, Long Beach, USA

**Keywords:** Myxoma, Cardiac mass, Echocardiogram, Sinus bradycardia, Pre-syncope, Coronary angiography, Mitral valve, Cardiology

## Abstract

**Background:**

Atrial myxomas account for approximately 50% of all primary cardiac tumors. The size, location, risk of embolic event, and involvement of other cardiac structures, are all factors that contribute to the wide range of presentation for cardiac myxomas. Patients with myxomas may remain asymptomatic, while others may report symptoms such as fatigue and fever, dyspnea, and syncope. It is important to recognize arrhythmias as an uncommon symptom of myxomas.

**Case presentation:**

We report a rare case of a 67-year-old man who presented with pre-syncopal episodes, symptomatic bradycardia, and night sweats found to have a 5.5 × 5.1 × 3 cm myxoma in the left atrium. During diastole the mass caused dynamic flow obstruction across the mitral valve. The patient underwent surgical resection of the mass given his symptomatology and risk of embolic events. Removal of the myxoma resulted in resolution of both pre-syncopal episodes and the patient’s sinus bradycardia.

**Conclusion:**

Atrial myxomas are a rare cause of pre-syncope and symptomatic bradycardia. It is important to have a clinical suspicion for atrial myxomas given early diagnosis and surgical intervention are key in improving the prognosis of these patients. This case also highlights the importance of taking into account the source of the myxoma’s blood supply in relationship to other cardiac structures, and further correlating these findings with clinical symptoms.

## Background

Cardiac masses are rare and have a wide range of clinical presentations depending on their location, which makes their diagnosis challenging. Cardiac masses can be primary or secondary depending on their origin, and further categorized as benign and malignant. Secondary, or metastatic tumors to the heart, are more common than primary cardiac tumors. Approximately 90% of primary cardiac masses are benign with the remaining 10% malignant. Myxomas are the most common benign cardiac mass and account for approximately half of all benign cardiac tumors [1]. Myxomas are most often found during the fourth to sixth decades of life, with a female predominance. The most common location of myxomas is the left atrium where they are found approximately 75% of the time. Less commonly, they may arise from the right atrium approximately 20% of the time, and around 5% of them are found in the ventricles [2]. The size, location, risk of embolic event, and involvement of other cardiac structures, are all factors that contribute to the wide range of presentation for cardiac myxomas. Patients with myxomas may remain asymptomatic, while others may report symptoms such as fatigue and fever, dyspnea, and syncope. Arrhythmias are uncommon with myxomas [3]. Here we present an unusual case of a 67-year-old man presenting with pre-syncope and symptomatic bradycardia due to a large left atrial myxoma.

### Case presentation

A 67-year-old male with chronic obstructive pulmonary disease (COPD), hypothyroidism, and asymptomatic sinus bradycardia, presented to the emergency department (ED) after experiencing a pre-syncopal episode. Earlier that week the patient developed sudden onset dizziness and light headedness while at dinner. Similar episodes over the past year were reported with worsening night sweats for several weeks. He denied headaches, vertigo, chest pain, shortness of breath, nausea, changes in vision or focal neurologic deficits. The lightheadedness lasted for about 30 s and then self-resolved, but he experienced persistent fatigue for a few hours afterwards. Prior to the episode of dizziness, he denied any notable changes with body position, flushing of the face/body, anxiety, and was not wearing any tight-fitting clothes. An electrocardiogram (ECG) showed sinus bradycardia and referred him to the emergency department for further evaluation.

**Fig. 1:**
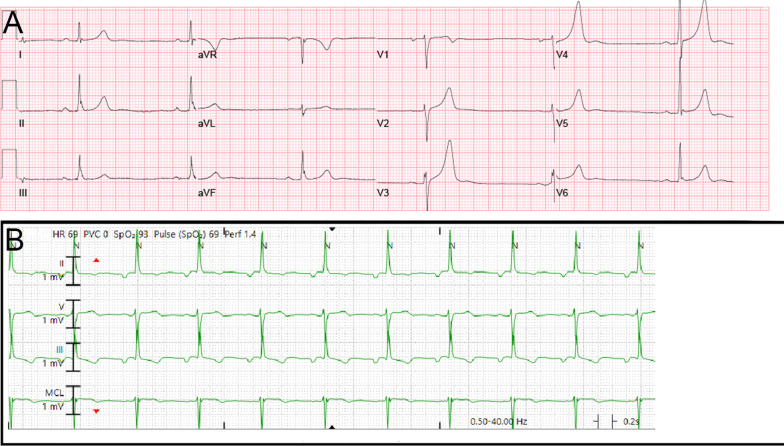
Electrocardiogram. **a** pre-operative electrocardiogram showing sinus bradycardia with a heart rate of 39 without ischemic changes. **b** post-operative electrocardiogram showing resolution of bradycardia with a heart rate of 69

In the emergency department, he was afebrile with a regular pulse of 39 bpm, blood pressure of 161/81, respiratory rate 18, and oxygen saturation was within normal limits on room air. Blood work was unremarkable. ECG showed sinus bradycardia, notched p-waves in limb lead II, and peaked T-waves in precordial leads V3, V4 and V5 with no evidence of heart block or ischemic changes (Fig. 1a). Chest x-ray showed no acute intrathoracic pathology, vascular congestion or pulmonary edema, but revealed a double density at the right heart border, consistent with left atrial enlargement. Physical exam revealed a regular heart rhythm without murmurs. Heart rate (HR) demonstrated an appropriate chronotropic response increasing from 40 to 60 s after 2 min of physical activity. No jugular venous distension or extremity edema was noted. The lungs were clear without rales or crackles. Neurological exam did not reveal any focal deficits, both motor and sensory function were intact bilaterally.

**Fig. 2:**
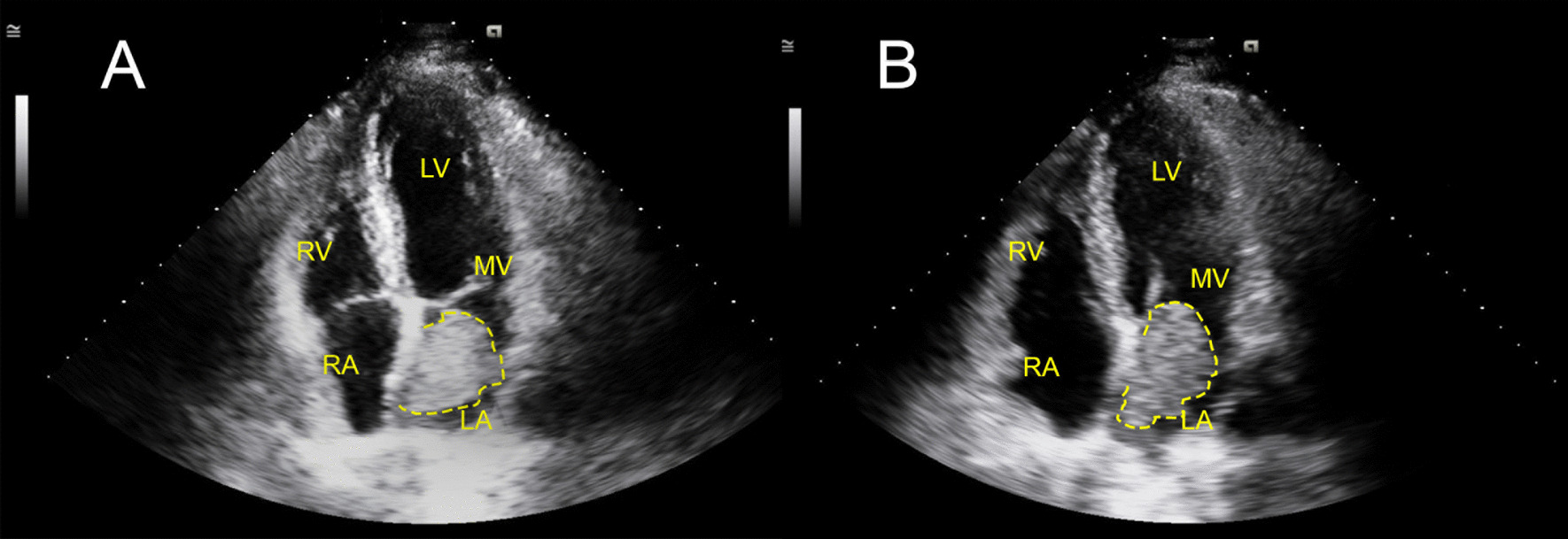
Transthoracic Echocardiogram During Systole and Diastole. **a** Left atrial myxoma attached to interatrial septum measuring approximately 3 cm × 4.8 cm. **b** Independently moving myxoma shown obstructing the mitral valve orifice during diastole. *LA* left atrium; *LV* left ventricle; *RA* right atrium; *RV* right ventricle

**Fig. 3:**
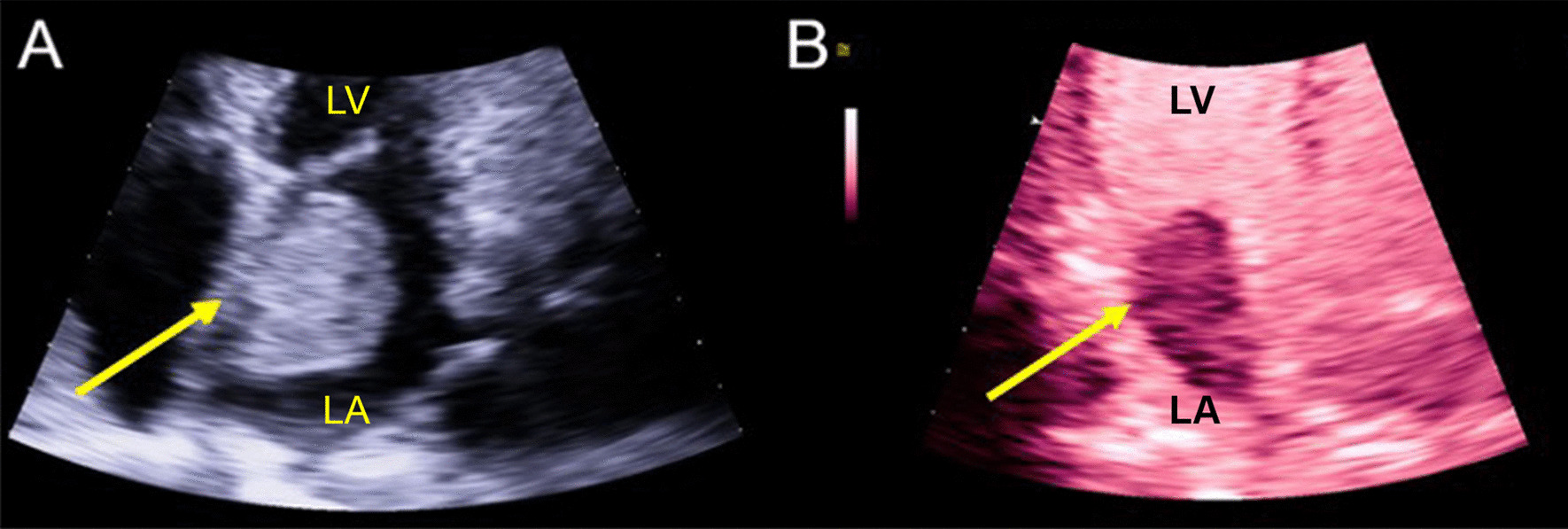
Myxoma as a Heterogeneous Hyperechoic Mass with Definity Enhanced Imaging. **a** Echocardiographic image showing (3 cm × 4.8 cm) heterogeneous hyperechoic mass in left atrium attached to the interatrial septum by a stalk (arrow). **b** Definity enhanced ultrasound of left atrial myxoma demonstrating heterogeneous echogenicity within the mass representative of vascularity

Transthoracic echocardiogram (TTE) showed a Left Ventricular Ejection Fraction (LVEF) of 60–65%, a positive bubble study and a mass in the left atrium estimated to be 3 cm × 4.8 cm, that was observed obstructing the mitral valve during diastole (Fig. 2a, b). The mass was a mobile, hyperechoic structure in the left atrium suggestive of cardiac myxoma or thrombus. Transthoracic echo further characterized the heterogeneous hyperechoic mass, showing hypoechogenic areas representing space neighboring the stalk (Fig. 3a). Definity enhanced contrast imaging of the myxoma similarly showed a heterogeneous mass which is representative of the mass’s vascularity, a characteristic finding which can help differentiate myxomas from thrombi, which tend to have a more homogeneous echogenicity (Fig. 3b).

### Outcome and follow-up

**Fig. 4:**
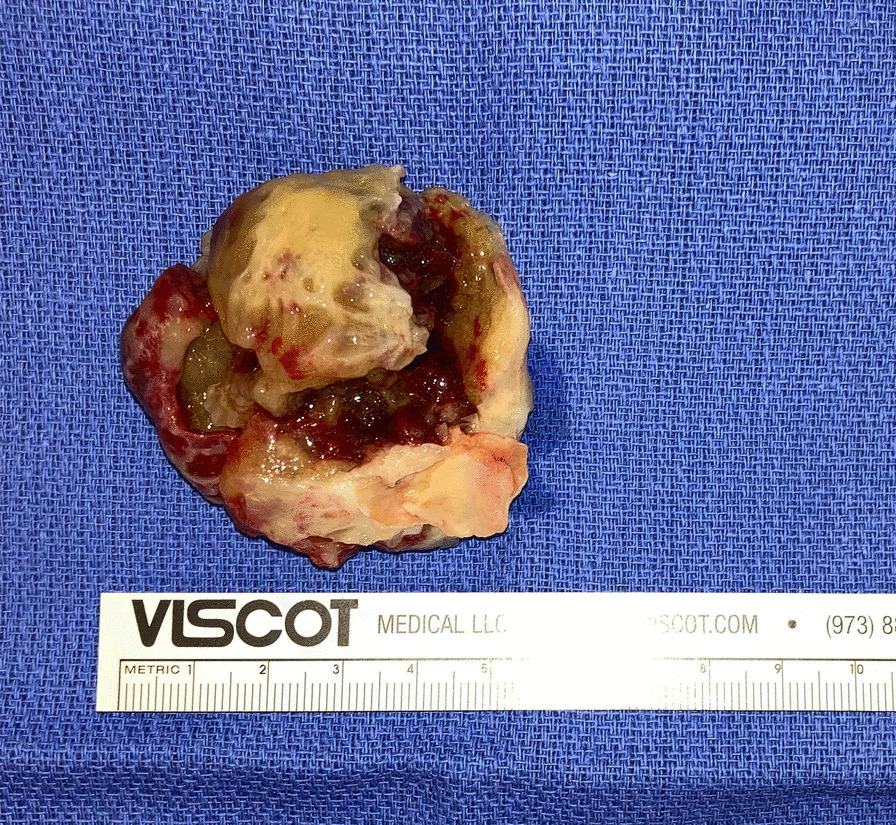
Resected Myxoma. Gross pathological specimen of myxoma that was resected with an estimated size of 5.5 × 5.1 × 3 cm, next to visual estimation of size

After the diagnosis of left atrial myxoma was made the patient was referred for surgical resection. Prior to surgery, coronary angiography demonstrated no significant coronary artery disease but did demonstrate tumor blushing consistent with myxoma. The patient then underwent left atrial mass resection and patent foramen ovale (PFO) repair. In the operating room, the atrial tumor was a large, solid, friable semi-mobile mass with a wide base stalk adherent to the left atrial septum, approximately 5.5 × 5.1 × 3 cm. The procedure was completed without any intraoperative complications. The gross specimen was dark-tan and red, with irregular borders and overall smooth surfaces (Fig. 4). The patient had an uncomplicated post-operative course and was discharged on post-op day 4. Interestingly upon follow-up one month after the myxoma was resected, the patient denied any additional pre-syncopal episodes and his bradycardia had resolved. Heart rate was 69 bpm 3 months after resection (Fig. 1b).

## Discussion and conclusions

**Fig. 5:**
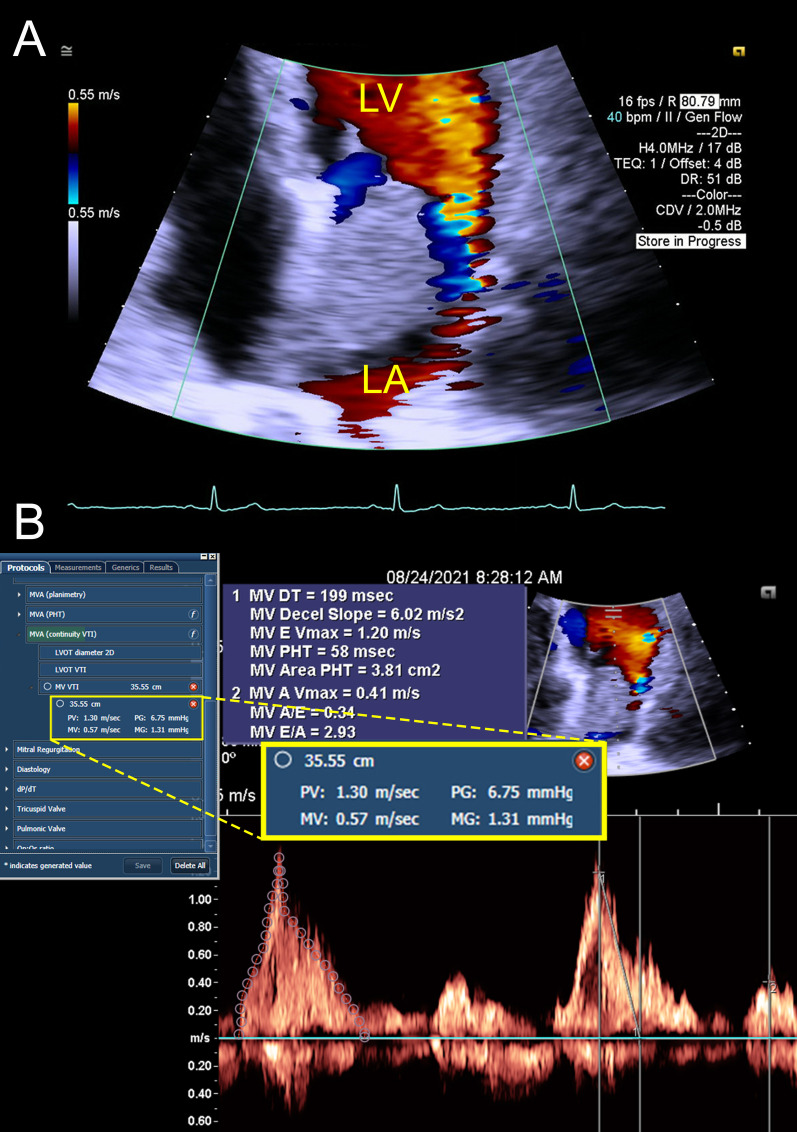
Doppler Flow and Calculated Mean Pressure Gradient Across Mitral Valve. **a** Doppler Image of Myxoma showing flow around the myxoma. **b** Mean pressure gradient across mitral valve measured in apical four chamber with Velocity Time Integral method, revealing Mean Pressure Gradient of 1.31 mmHg

While patients are frequently evaluated for causes of pre-syncope and syncope, intracardiac masses represent an unusual and rare culprit. In this case the left atrial myxoma was causing intermittent flow obstruction, mimicking severe mitral stenosis when it descended into the plane of the mitral valve (MV) annulus during diastole leading to the patient’s pre-syncopal event. Using velocity time integral method, the calculated mean pressure gradient across the MV was measured to be 1.31 mmHg (Fig. 5a, b). When compared with mitral valve stenosis, this was consistent with insignificant obstruction (EAE/ASE classification: mild < 5 mm Hg, moderate 5–10 mm Hg and severe > 10 mmHg) [4]. It is important to recognize that the echocardiography was captured while the patient was hospitalized, an interval where the patient did not have additional pre-syncopal or syncopal events after being adequately volume resuscitated. Therefore, it is possible that during the patient’s initial pre-syncopal event, there was more severe blood flow obstruction and a higher mean gradient pressure across the MV, which was likely exacerbated by a combination of dehydration and body positioning. If the patient was experiencing a hypovolemic state from dehydration, this would lead to decreased pre-load and subsequently exacerbating the myxoma’s obstruction of the mitral valve. Additionally given the patient presented with a heart rate of 39, an episode of marked bradycardia could also serve as either the cause or a contributing factor to the pre-syncopal episode.

**Fig. 6:**
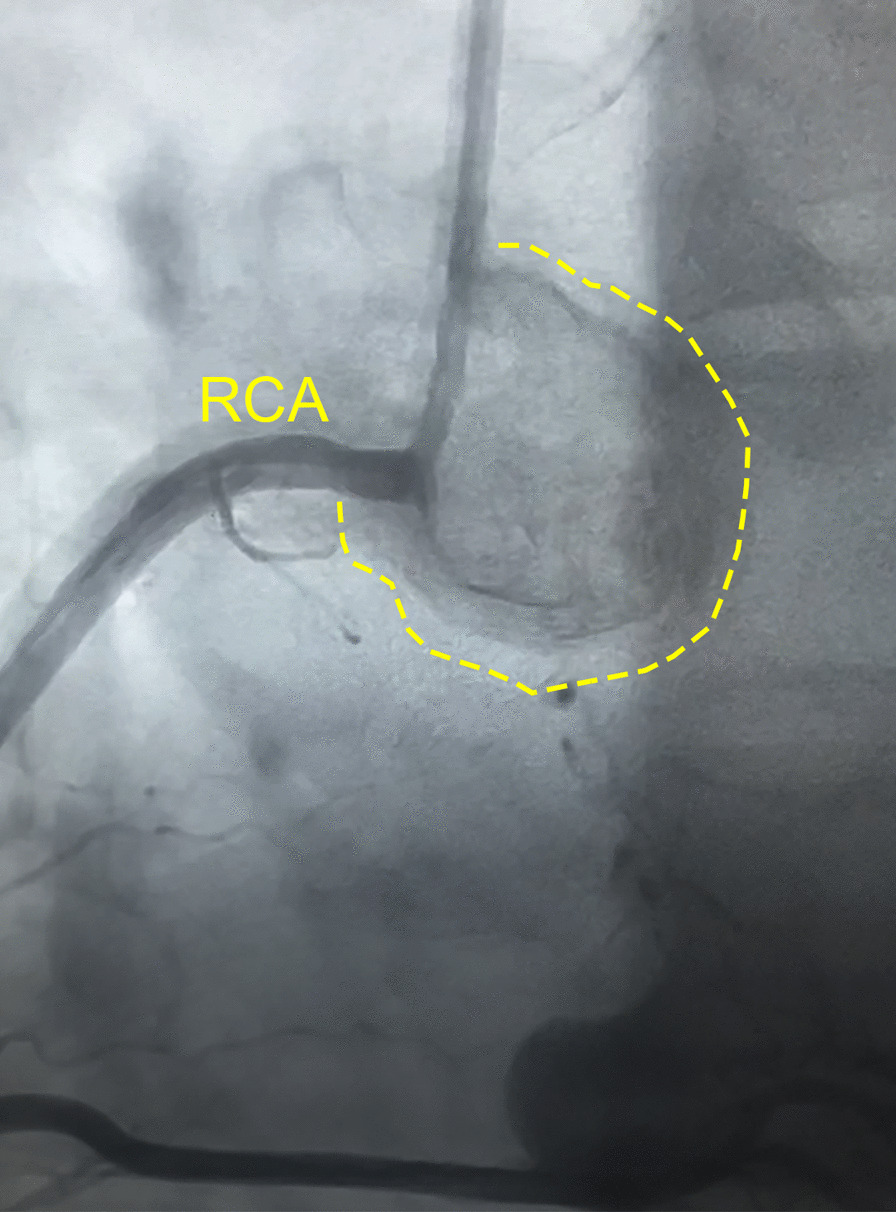
Coronary Angiography with Tumor Blushing. Angiography of RCA showing perfusing vessels to the left atrial myxoma with tumor blushing. *RCA* right coronary artery

Another unique aspect of this case is the resolution of the patient’s bradycardia after surgical removal of the myxoma. Conduction and rhythm disorders are rare symptoms from cardiac myxomas, occurring in less than 10% of cases [3, 5]. The etiology by which myxomas cause conduction delays is unclear, as these tumors are typically non-invasive, form on the endocardium and rarely grow to reach the epicardium. However, the presence of a vascular supply to a myxoma can be associated with rare presentations such as angina and sick sinus syndrome due to blood shunting and nodal ischemia [6]. Given myxomas typical posterior location in the left atria, they are commonly supplied by the right coronary artery (RCA) leading to decreased blood flow to the sinus node [6]. In our case, the patient had an angiogram showing perfusing vessels to the myxoma which may have led to decreased blood supply to the sinus node resulting in bradycardia (Fig. 6). Sinus bradycardia is an unusual presentation of left atrial myxomas, and in literature these cases remain rare [7, 8].

This case illustrates the importance of having a high clinical suspicion and considering myxomas when determining the cause of symptomatic bradycardia and pre-syncope. The left atrial myxoma received its blood flow from the RCA, which also provides blood to the sinoatrial node. Although the pathophysiological mechanism behind left atrial tumor sick sinus syndrome remains a topic for further investigation, in our case it is possible that the sinus bradycardia is a result of shunting of blood away from the sinoatrial node and towards the myxoma. In support of this, after the left atrial myxoma was removed, the patient’s sinus bradycardia resolved.

## Data Availability

The datasets and imaging used and/or analyzed during the current study are available from the corresponding author on reasonable request.

## Abbreviations

COPD: Chronic obstructive pulmonary disease
ED: Emergency department
EKG: Electrocardiogram
HR: Heart rate
TTE: Transthoracic echocardiogram
LVEF: Left ventricular ejection fraction
PFO: Patent foramen ovale
MV: Mitral valve
